# Molecular regulation of auditory hair cell death and approaches to protect sensory receptor cells and/or stimulate repair following acoustic trauma

**DOI:** 10.3389/fncel.2015.00096

**Published:** 2015-03-31

**Authors:** Christine T. Dinh, Stefania Goncalves, Esperanza Bas, Thomas R. Van De Water, Azel Zine

**Affiliations:** ^1^University of Miami Ear Institute, University of Miami Miller School of MedicineMiami, FL, USA; ^2^Integrative and Adaptive Neurosciences, Aix-Marseille Université, CNRS, UMR 7260Marseille, France; ^3^Faculty of Pharmacy, Biophysics Department, University of MontpellierMontpellier, France

**Keywords:** trauma, cochlea, auditory hair cells, apoptosis, inflammation, necrosis, otoprotection, repair

## Abstract

Loss of auditory sensory hair cells (HCs) is the most common cause of hearing loss. This review addresses the signaling pathways that are involved in the programmed and necrotic cell death of auditory HCs that occur in response to ototoxic and traumatic stressor events. The roles of inflammatory processes, oxidative stress, mitochondrial damage, cell death receptors, members of the mitogen-activated protein kinase (MAPK) signal pathway and pro- and anti-cell death members of the Bcl-2 family are explored. The molecular interaction of these signal pathways that initiates the loss of auditory HCs following acoustic trauma is covered and possible therapeutic interventions that may protect these sensory HCs from loss via apoptotic or non-apoptotic cell death are explored.

## Introduction

Auditory hair cells (HCs) are important for the conversion of acoustic sound energy into electrical impulses that travel to the auditory centers of the brain for hearing. Sensorineural hearing loss (SNHL) is a form of hearing impairment that occurs most commonly from damagedHCs within the cochlea; it is a prevalent disability, affecting one in five people and more than 48 million Americans (Lin et al., 2011). There are numerous causes of acquired SNHL. Some of these etiologies include viral infections, platinum-based chemotherapeutic agents, aminoglycoside antibiotics, acoustic trauma, labyrinthine concussion, cochlear hypoxia, radiation exposure, cochlear implant electrode insertion trauma, and meningitis (Kuhn et al., 2011). Genetic mutations can also cause structural or physiologic abnormalities within the cochlea and impair cochlear homeostasis. These insults to the inner ear can promote cell death of auditory HCs and hearing loss.

Several signaling cascades are activated following an insult to the cochlea; these pathways can be pro-inflammatory, pro-death, and even pro-survival. The signaling cascades that occur on a cellular and a molecular level are highly complex and in many ways entwine; there is significant cross communication between pathways and it is the culmination of all of these activities that tilt the pendulum of cell survival and cell death in one direction or the other. Although there has been great progress in understanding the pathways of cell death in auditory HCs, several pro-death concepts are still speculative and extrapolated from studies of other cell types. As research in necrobiology of the inner ear progresses, these pathways specific to auditory HC death will be more stream-lined and refined. This review will discuss general concepts and pathways in apoptosis and necrosis that contribute to the understanding of key events in acoustic trauma. Several drug therapies take advantage of key events that occur following acoustic injury by targeting, promoting, or inhibiting different pathways that favor cell survival.

## Cell Death

**Figure 1:**
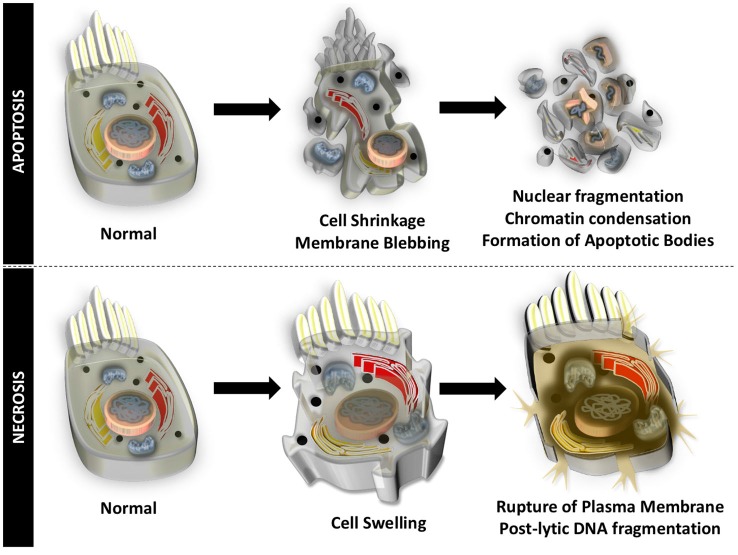
**Apoptotic and necrotic cell death**. Apoptosis is an organized form of programmed cell death that is characterized by shrinkage of the cell body, blebbing of the cell membrane, chromatin condensation, fragmentation of the nucleus, cleavage of chromosomal DNA, and division into numerous cell fragments (apoptotic bodies).Necrosis occurs when the volume of the cell increases, organelles swell, the plasma membrane ruptures, intracellular contents spill into the extracellular matrix, and there is postlytic DNA fragmentation.

Auditory HCs undergo cell death through apoptosis and necrosis, in response to different insults to the inner ear. Apoptosis is an organized form of programmed cell death that is characterized by blebbing of the cell membrane, shrinkage of the cell body, fragmentation of the nucleus, condensation of the chromatin, and cleavage of chromosomal DNA. The cell is then divided into numerous fragments called apoptotic bodies that are disposed by phagocytosis (Figure 1). Particular to HCs undergoing apoptotic cell death, mitochondria swell, stereocilia are disrupted and lost, cuticular plates shrink, and chromatin condensation occurs in the nucleus, prior to disruption of junctional complexes and extrusion of auditory HCs (Hirose et al., 2004). Necrosis, originally thought to be an uncontrolled process, is another form of cell death that appears to be highly regulated. During necrotic cell death, the cell volume increases, the organelles swell, and the plasma membrane ruptures. There is spillage of intracellular contents in the extracellular space and postlytic DNA fragmentation occurs (Figure 1). Necrosis of auditory HCs can occur in concert with mechanisms of apoptosis. Most of the molecular and cellular mechanisms responsible for apoptosis and necrosis in auditory HCs originate in studies of other cell types. A review of general concepts in apoptosis and necrosis and stress signaling is offered below.

## Apoptosis

Apoptosis can occur through two different signaling cascades, called the intrinsic [mitochondrial death] and extrinsic [death receptor, DR] pathways. These two particular pathways can interweave and can promote synergism in cell death in many contexts (Roy and Nicholson, 2000).

### Intrinsic Pathway

**Figure 2:**
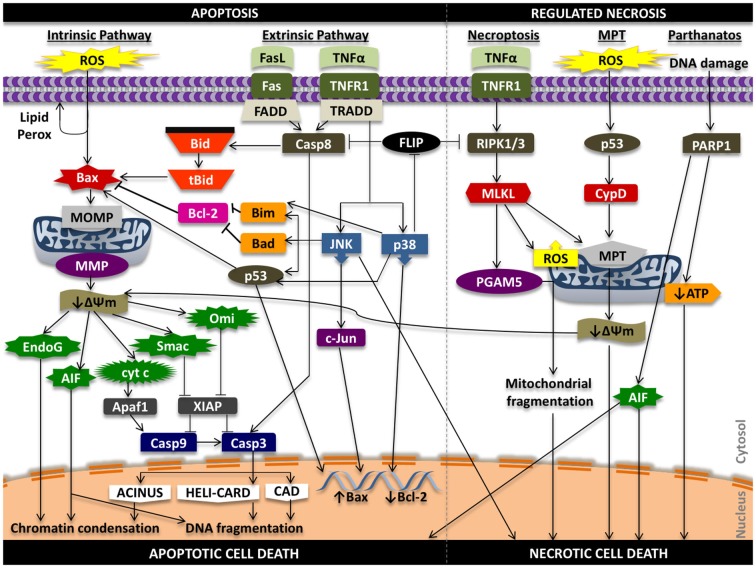
**Signaling in apoptosis and regulated necrosis**. ROS can promote lipid peroxidation of the phosopholipid membrane, proteolytic degradation, and mitochondrial and cellular DNA damage that can propagate the apoptotic effects of oxidative stress. Oxidative stress can activate pro-apoptotic Bax protein that initiates damage to the outer mitochondrial membrane (i.e., pore formation) that results in release of pro-death proteins from the mitochondria into the cytosol that act in caspase-independent and caspase-dependent mechanisms to induce chromatin condensation, DNA fragmentation and intrinsic apoptotic cell death. Ligands such as Fas ligand (FasL) and tumor necrosis factor alpha (TNFα) can promote extrinsic programmed cell death through caspase-8 activation and downstream caspase-3 activity, while also triggering intrinsic apoptosis through truncation of Bid (tBid). Tumor necrosis factor receptor type 1 (TNFR1) signaling can induce phosphorylation and activation of p38 and JNK that can lead to Bax-mediated mitochondrial cell death and phosphorylation of transcription factors that increase pro-death and reduce pro-survival gene expression. Activation of TNFR1 can also initiate a RIPK1 and RIPK3-dependent form of regulated necrosis (necroptosis) through regulation of PGAM5, ROS, and the mitochondrial permeability transition (MPT). MPT-mediated regulated necrotic cell death can be triggered following oxidative stress; downstream signaling results in activation of cyclophilin D (CypD), increased permeability of the inner mitochondrial membrane, and mitochondrial swelling and rupture that promotes necrosis. Alterations in the transmembrane potential of the mitochondria can also induce intrinsic cell death. Lastly, hyper-activation of PARP1 from persistent DNA damage that can inhibit mitochondrial ATP synthesis and promote mitochondrial release of AIF to initiate necrotic form of cell death. AIF, in this situation, can promote both apoptosis and necrosis. ΔΨm, mitochondrial membrane potential; Casp3, caspase-3; Casp8, caspase-8; Casp9, caspase-9; MMP, mitochondrial membrane permeability; MOMP, mitochondrial outer membrane permeability.

In the intrinsic pathway, a stress signal initiates a disturbance between the pro- and anti-apoptotic proteins of the Bcl-2 family that promote supramolecular openings or activation of mega-channels in the outer membrane of the mitochondria (Marzo et al., 1998; Kuwana et al., 2002). In particular, Bcl-2-like protein 4 (Bax) activation is a key regulator of this phenomenon. This results in the release of pro-death proteins from the intermembrane space of the mitochondria into the cytosol. These regulatory mitochondrial proteins that are liberated into the cytoplasm can activate both caspase-dependent and caspase-independent cell death pathways. These proteins include cytochrome *c* (cyt *c*), endonuclease G (EndoG), apoptosis inducing factor (AIF), second mitochondria-derived activator of caspases/direct inhibitor of apoptosis protein binding protein with low pI (Smac/DIABLO), and mammalian homolog of bacterial high temperature requirement protein A2 (Omi/HtrA2; Figure 2).

When released into the cytoplasm, cyt *c* binds with apoptotic protease-activating factor-1 (Apaf-1) and recruits pro-caspase-9 to form an apoptosome (Cain et al., 2000). This apoptosome can induce caspase-3 dependent apoptosis (Bratton et al., 2001). Subsequently, caspase-3 initiates apoptosis by promoting DNA fragmentation through caspase-activated DNase (CAD), chromatin condensation via apoptotic chromatin condensation inducer in the nucleus (ACINUS), and acceleration of DNA degradation through cleavage of cytosolic helicase with an N-terminal caspase-recruitment domain (HELI-CARD; Liu et al., 1997; Enari et al., 1998; Sahara et al., 1999; Kovacsovics et al., 2002).

EndoG is a mitochondrion-specific nuclease that translocates into the nucleus and works in concert with exonucleases and DNAse I to ensure efficient nucleosomal fragmentation of DNA, independent of caspase activation (Li et al., 2001; Widlak et al., 2001). Similar to EndoG, AIF is also a caspase-independent death effector; once released into the cytosol, AIF migrates into the nucleus to stimulate chromatin condensation and large scale DNA fragmentation (Lorenzo et al., 1999; Daugas et al., 2000).

Smac and Omi/HtrA2 are similar because both promote caspase-dependent apoptosis by binding and inhibiting X-linked inhibitor of apoptosis protein (XIAP). XIAP is a cytosolic protein that has three baculoviral inhibitory repeat (BIR) domains—BIR1 and BIR2 specifically bind and inhibit caspase-3 and -7, while BIR3 is a specific inhibitor of caspase-9 (Deveraux et al., 1999). Smac functions by neutralizing the caspase-inhibiting properties of XIAP, thereby promoting caspase-3, -7, and -9 activations (Chai et al., 2000, 2001). Similar to Smac, Omi/HtrA2 competes with caspase -3, -7, and -9 for XIAP binding and therefore promotes caspase-dependent cell death (Suzuki et al., 2001; Hegde et al., 2002). However, Omi/HtrA2 is a ubiquitously expressed mitochondrial serine protease that can also promote apoptosis through caspase-independent activity through alternate mechanisms that rely on its serine protease properties (Li et al., 2002).

### Extrinsic Death Pathway

The extrinsic cell death pathway is intricate and involves several molecular interactions that occur in succession: (1) binding of a death ligand to its complementary receptor; (2) recruitment of adaptor molecules such as FAS-associated death domain protein (FADD) and tumor necrosis factor receptor type 1-associated death domain protein (TRADD); (3) binding, dimerizing, and activation of initiator caspase-8 and -10; and (4) formation of a death-inducing signaling complex (DISC; Itoh and Nagata, 1993; Tartaglia et al., 1993; Chinnaiyan et al., 1995; Hsu et al., 1995; Nagata, 1999; Fischer et al., 2006). DISC is a multi-protein complex that subsequently cleaves and promotes executioner caspase-3 and -7 activities to promote programmed cell death (Figure 2).

The most well recognized and studied death ligands include TNFα and FasL or CD95L. Their complementary receptors are TNFR1, also known as p55 or CD120a and Fas receptor (FasR, also referred to as CD95 or apoptosis antigen 1, APO-1), respectively (Itoh and Nagata, 1993; Tartaglia et al., 1993). Other DRs that have been described include TNF-like receptor apoptosis mediating protein (TRAMP, also called DR3, APO-3), TNF-related apoptosis inducing ligand receptors-1 (TRAIL-R1 or DR4) and -2 (TRAIL-R2, also named DR5 and APO-2), and DR6 (Bodmer et al., 1997; Guicciardi and Gores, 2009).

Initiators caspase-8 and caspase-10 can cleave and activate effector caspase-3 to initiate programmed cell death (Ng et al., 1999; Wang et al., 2001; Fischer et al., 2006). Caspase-8 can also promote effector caspase-7 activity. In addition, both caspase-8 and caspase-10 can cleave Bcl-2 homology 3 interacting domain death agonist (BID) into truncated BID (tBID) that triggers mitochondrial cell death pathways mediated by Bax and Bcl-2 homologous antagonist killer (Bak) activation (Chandler et al., 1998; Li et al., 1998; Luo et al., 1998; Korsmeyer et al., 2000; Kandasamy et al., 2003; Milhas et al., 2005). Bax and Bak are pro-death proteins that belong to the Bcl-2 family of proteins that can stimulate mitochondrial release of pro-apoptotic proteins such as cyt *c* and Smac. There are likely other levels of modulation between the intrinsic and extrinsic pathways of apoptosis that have not yet been discovered.

## Necrosis

Mechanisms of regulated necrosis include necroptosis and MPT. Parthanatos, ferroptosis and pyroptosis have also been described as mechanisms of non-apoptotic cell death; however, they may not represent distinct forms of necrosis (Galluzzi et al., 2012; Vanden Berghe et al., 2014; Figure 2).

### Necroptosis

Receptor interacting protein kinases (RIPK) are important regulators of cell survival and cell death. Necroptosis refers to a RIPK3-dependent molecular cascade of events that promotes regulated necrotic cell death (Galluzzi et al., 2012). Necroptosis and apoptosis share similar upstream signaling elements and regulator molecules such as Fas-associated death domain-like interleukin-1β-converting enzyme-like inhibitory proteins (FLIP) and cellular inhibitors of apoptosis proteins 1 and 2 (cIAP1 and cIAP2; McComb et al., 2012; Silke and Strasser, 2013). When caspase-8 is inhibited by genetic manipulation or pharmacologic therapies, RIPK3 is recruited to RIPK1 to form a necrosis-inducing complex (necrosome) that phosphorylates pseudokinase mixed lineage kinase domain-like protein (MLKL) and engages in downstream activities that lead to RIPK1/RIPK3 dependent necroptosis (He et al., 2009; Zhang et al., 2009; Vandenabeele et al., 2010). The events that occur downstream to promote necroptosis are controversial, convoluted, and poorly characterized, but may include production of mitochondrial reactive oxygen species (ROS), activation of mitochondrial phosphatase, i.e., PGAM5, and induction of MPT (Wang et al., 2012a,b; Marshall and Baines, 2014). PGAM5 is a mitochondrial protein phosphatase that can activate dynamin related protein 1 (Drp1) and its GTPase activity, which can stimulate fragmentation of mitochondria and execution of necrosis (Wang et al., 2012a,b).

### Mitochondrial Permeability Transition

The MPT refers to an abrupt increase in the permeability of the inner mitochondrial membrane to small solutes that lead to osmotic influx of water into the mitochondrial matrix, mitochondrial swelling and rupture of the outer mitochondrial membrane (Tsujimoto and Shimizu, 2007). Although it is not well characterized, cyclophilin D (CypD; also known as peptidylprolyl isomerase F) is believed to be crucial in the formation of the permeability transition pore complex associated with MPT-dependent necrosis (Li et al., 2004; Baines et al., 2005; Nakagawa et al., 2005). Interestingly, the shift in the transmembrane potential in MPT can also initiate intrinsic cell death by halting the bioenergetics and redox functions of the mitochondria and initiating release of pro-apoptotic mitochondrial proteins into the cytosol (Marchetti et al., 1996; Scarlett and Murphy, 1997; Brenner and Grimm, 2006; Kroemer et al., 2007).

### Other Forms of Non-Apoptotic Cell Death

Parthanatos is a poly(ADP-ribose) polymerase 1 (PARP1) dependent form of non-apoptotic death. PARP1 is recruited to sites of DNA damage where it is able to catalyze ADP ribosylation of various proteins and histones and generate negative charges by overconsumption of nicotinamide adenine dinucleotide (NAD) during the ADP ribosylation process. Ultimately, other factors important for DNA repair are recruited. However, when PARP1 becomes hyper-activated in situations of persistent DNA damage, there is a depletion of NAD and inhibition of mitochondrial ATP synthesis, which is essential in ATP-dependent apoptosis pathways (Yu et al., 2006; Wang et al., 2011). Overactivation of PARP-1 can lead to unregulated synthesis of poly (ADP-ribose) (PAR) that can bind to and initiate release of AIF into the cytoplasm. PARP-1 mediated AIF expression can promote DNA condensation and widespread cell death that is independent of caspases and distinct from apoptosis (Andrabi et al., 2008). There is controversy whether parthanatos represents a form of regulated necrosis or is a distinct entity.

Ferroptosis refers to an iron-dependent form of non-apoptotic cell death that is morphologically, biochemically, and genetically different from both apoptosis and necrosis (Dixon et al., 2012). Pyroptosis refers to caspase-1 dependent cell death that exhibits a spectrum of morphotypes that range from necrosis to purely apoptosis (Kepp et al., 2010). Caspase-1 is activated through a supramolecular complex called a pyroptosome or inflammasome and is thought to cause pore formation in the plasma membrane that leads to osmotic cell lysis (necrosis) and activation of caspase-7 (apoptosis) (Fink and Cookson, 2006; Fernandes-Alnemri et al., 2007; Lamkanfi et al., 2008). The elements behind regulated necrosis are still unclear; however, research in this topic has become more and more prevalent especially in the context of cancer research.

## Auditory Hair Cell Death

Depending on the cochlear insult, various elements of the apoptotic and necrotic cell death pathway are activated (Op de Beeck et al., 2011; Figure 1). In acoustic trauma, loss of auditory HCs occur through direct mechanical injury and activation of apoptosis and necrosis related pathways (Saunders et al., 1985; Pirvola et al., 2000; Yang et al., 2004). Intense noise exposure can displace the tympanic membrane, vibrate the ossicles, and create large displacements of the basilar membrane that can sheer and injure stereocilia, auditory HCs, and supporting cells of the organ of Corti important for hearing. Downstream stress signaling from acoustic trauma are activated, including expression of free radicals and pro-inflammatory factors that trigger both apoptotic and necrotic cell death (discussed in subsequent sections). Following intense noise exposure Bax activation promotes mitochondrial release of pro-apoptotic factors (i.e., cyt *c*, AIF, and EndoG), caspase-3 activation, and intrinsic cell death (Nicotera et al., 2003; Yamashita et al., 2004a,b; Han et al., 2006; Wang et al., 2007a,b). Acoustic trauma also stimulates TNFR1 and Fas DR signaling that activates downstream pathway leading to caspase-8 activity and extrinsic apoptotic cell death (Nicotera et al., 2003; Fujioka et al., 2006; Jamesdaniel et al., 2011). Although the molecular mechanisms are still unclear, necrosis of outer HCs occur early following acoustic trauma and persist weeks after acoustic trauma (Yang et al., 2004; Hu et al., 2006). Lastly, intense noise exposure can also promote loss of auditory HCs through HC extrusion (Cotanche et al., 1997).

Although there are several drugs that can injure auditory HCs, the most commonly encountered ototoxic drugs are aminoglycosides and cisplatin. In brief, aminoglycoside antibiotics, such as gentamicin, amikacin, kanamycin, and neomycin, can initiate apoptotic cell death in auditory HCs and vestibular HCs. In particular, the outer HCs of the basal turn are the most vulnerable to aminoglycoside ototoxicity. The most predominant form of cell death is apoptosis, however necrotic features are also seen following exposure to aminoglycosides (Jiang et al., 2006). Aminoglycosides can induce mitochondrial apoptotic cell death and DNA fragmentation through oxidative stress, Bax activation, mitochondrial release of cyt *c* and caspase-3 activity (Mangiardi et al., 2004; Matsui et al., 2004; Coffin et al., 2013). Evidence for the role of caspase-8 and extrinsic apoptosis is aminoglycoside ototoxicity is not strong (Tabuchi et al., 2007; Ding et al., 2010).

Cisplatin is a platinum-based chemotherapeutic drug used to treat cancer and can promote ototoxicity, nephrotoxicity, and even neurotoxicity. Cisplatin predominantly affects the outer HCs of the basal turn of the cochlea; spiral ganglion neurons are affected by this drug. Similar to aminoglycosides, cisplatin stimulates free radical production in the cochlea that leads to Bax activation, mitochondrial release of cyt *c*, caspase activation, and intrinsic apoptotic cell death (Rybak et al., 2007). Unfortunately, regulated necrosis and potential pathways associated with this form of cell death are still unclear in the field of cell biology. The contribution of pathways described in regulated necrosis is still speculative in cochlear injury. Progress in necrobiology research can improve our understanding of necrosis in auditory HCs.

## Stress Signaling in Auditory Hair Cell Death

There are several signaling pathways that are involved in apoptosis and necrosis of auditory HCs, including (1) expression of extracellular pro-inflammatory cytokines such as tumor necrosis factor alpha (TNFα) and recruited of neutrophils and macrophages to the cochlea; and (2) generation of oxidative stress in the form of ROS and reactive nitrogen species (RNS) such as superoxide (${\text{O}}_{\text{2}}^{\text{.−}}$), peroxynitrite (ONOO^−^), and hydroxyl (${\text{OH}}^{\text{.}}$) radicals (Figure 2). These stress signals can activate intrinsic and extrinsic apoptotic cell death of auditory HCs as well as initiate molecular mechanisms for necrosis. Although signaling mechanisms discussed are extrapolated from studies investigating the effects of various cochlear insults on apoptosis and necrosis in auditory HCs and studies of other cell types, many of these pathways are relevant and expressed following acoustic trauma. Studies pertaining to stress signaling in the cochlea are bolded in subsequent sections.

### Inflammatory Cytokines and Chemokines

Several cytokines and chemokines are upregulated in the cochlea following an inner ear insult. TNFα is one of the most well studied and possibly most important cytokines as it possesses pro-inflammatory properties and can also initiated cell death of auditory HCs. TNFα is expressed within the cochlea during acoustic trauma, cisplatin ototoxicity, aminoglycoside exposure, electrode insertion trauma, autoimmune inner ear disease, as well as various other traumas to the inner ear (Ichimiya et al., 2000; Satoh et al., 2002, 2003; Aminpour et al., 2005; Zou et al., 2005; Fujioka et al., 2006; Van Wijk et al., 2006; So et al., 2007; Bas et al., 2009, 2012a,b).

Following a cochlear insult, the stria vascularis and spiral ligament express and release TNFα, which promotes leukocyte migration through the spiral modiolar vein and tributaries (Keithley et al., 2008). TNFα also stimulates expression of other cytokines, chemokines, and adhesion molecules such as interleukin (IL)-1β, IL-6, monocyte chemoattractant protein-1 (MCP-1), macrophage inflammatory protein-2 (MIP-2), soluble intercellular adhesion molecule-1 (siCAM-1), vascular cell adhesion molecule-1 (VCAM-1), intercellular adhesion molecule-1 (ICAM-1), and vascular endothelial growth factor (VEGF; Yoshida et al., 1999; Ichimiya et al., 2000, 2003; Maeda et al., 2005). These inflammatory mediators can promote migration and adhesion of inflammatory cells such as neutrophils, monocytes, macrophages, lymphocytes, eosinophils, basophils, and even natural killer cells. (Taub et al., 1995; Proost et al., 1996; Mutsaers et al., 1997; Deshmane et al., 2009). These inflammatory cells are themselves a major source of cytokines and chemokines, creating a positive feedback loop that propagates and intensifies the inflammatory process.

In particular, expression of TNFα can promote neutrophil migration, adhesion, and generation of superoxide free radicals in the cochlea (Tsujimoto et al., 1986; Figari et al., 1987). Neutrophils, one type of polymorphonuclear white blood cell, are among the first immune cells to arrive in response to inflammation. They relesase a number of pro-inflammatory cytokines that stimulate migration of other immune cells, such as monocytes (Mutsaers et al., 1997). In response to local cytokines, monocytes will differentiate into M1 and M2 macrophages. M1 macrophages are primarily involved in clearance of injured cells; they can secrete inflammatory factors, stimulate inducible nitrous oxide synthetase (iNOS) activity, and generate oxidative stress in the form of ROS and RNS that can diffuse into surrounding tissue. M2 macrophages are known to release several growth factors from the transforming growth factor-beta (TGF-β) family of proteins, which are important for initiation of a fibroproliferative response, wound healing, and tissue repair (Mahdavian Delavary et al., 2011).

Not only is TNFα a leukocyte attractant; extracellular TNFα can bind to tumor necrosis factor receptor 1 (TNFR1) on the cell surface of auditory HCs and initiate a signaling cascade that can lead to cell death (Dinh et al., 2008a,b, 2011, 2013; Haake et al., 2009; Figure 2). Adaptor protein TRADD, receptor interacting protein (RIP), and FADD are recruited along with caspase-8 to form a DISC that cleaves and activates caspase-3 and -7 and triggers extrinsic apoptosis. TNFα-mediated Bax activation and intrinsic programmed cell death also occurs, likely through truncation of Bid.

The binding of TNFα to TNFR1 can also promote formation of another signaling complex consisting of TNFR1, TRADD, RIP, and TNF receptor-associated factor 2 (TRAF2) that promotes mitogen-activated protein kinase (MAPK) and nuclear factor kappa B (NFkB) signaling (Shen and Pervaiz, 2006). NFkB is a transcription factor that can mediate cellular inflammation, proliferation, and apoptosis. When activated, NFkB can translocate into the nucleus of an affected cell and activate transcription of several genes that are pro-inflammatory, pro-survival and pro-death, depending on the cell type (Pahl, 1999). TNFα initiates NFkB signaling in an attempt to block cell death by up regulation of pro-survival genes Bcl-2 and Bcl-xL in auditory HCs (Haake et al., 2009; Dinh et al., 2011).

High levels of IL-1beta (β) have also been detected in OC and cochlea following various inner ear challenges such as exposure to gentamicin, electrode insertion trauma, and autoimmune responses mediated by inner ear tissues (Pathak et al., 2011; Bas et al., 2012a,b). This cytokine can promote formation of a pyroptosome or inflammasome that mediates caspase-1 dependent cell death via pyroptosis (Brough and Rothwell, 2007). As discussed earlier, caspase-1 activation can promote apoptosis by inducing caspase-7 activity or necrosis through pore formation in the cell membrane and cell lysis through an osmotic imbalance (Fink and Cookson, 2006; Fernandes-Alnemri et al., 2007; Lamkanfi et al., 2008). In summary, TNFα expression in the cochlea can promote a robust inflammatory response and activation of downstream pro-apoptotic cell signaling that can promote cell death of auditory HCs. Blocking TNFα expression and associated signaling cascades may mitigate the inflammatory and pro-death responses in the cochlea. In particular, glucocorticoids have been shown to reduce inflammatory cell trafficking as well as inhibit TNFα-mediated downstream pathways of cell death (Dinh et al., 2008a,b; Haake et al., 2009). Steroids can also activate NFkB signaling that promotes cell survival pathways following TNFα-induced death of auditory HCs (Dinh et al., 2011). Inhibition of Bax expression by short interfering RNA can also abrogate TNFα-mediated HC loss (Dinh et al., 2013).

### MAPK Signaling

**Figure 3:**
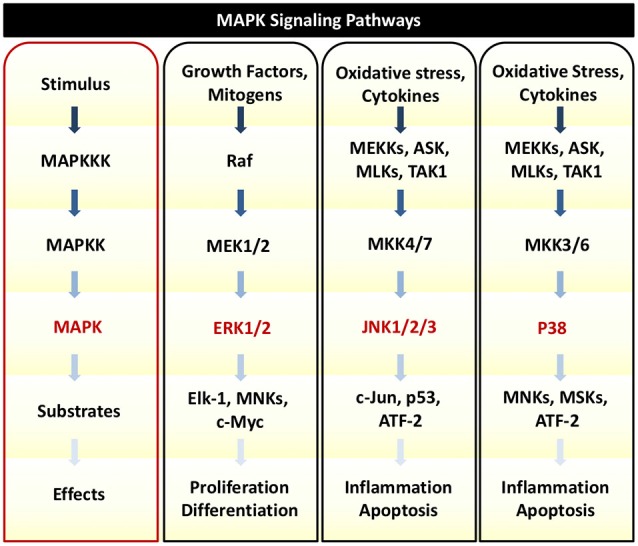
**MAPK Signaling**. There are three tiers of protein kinases that comprise each family of MAPKs: (1) MAPK; (2) MAPK kinase (MAPKK); and (3) MAPKK kinase (MAPKKK). Following a specific stimulus, MAPKKK phosphorylates and activates MAPKK, which then phosphorylates and activates MAPK to then phosphorylate target substrates that can regulate cellular proliferation, survival, inflammation, and cell death. The three main classes of MAPK include ERK1/2, JNK1/2/3, and p38. Oxidative stress and cytokines such as TNFα can trigger JNK and p38 to phosphorylate downstream transcription factors that initiate pro-death and pro-inflammatory signaling and gene transcription.

In response to TNFα, auditory HCs can initiate MAPK signaling pathways. There are several distinct groups of MAPKs; however, the most extensively studied MAPKs are extracellular signal-regulated kinases (ERK) 1 and 2 (ERK1/2), c-Jun N-terminal kinases (JNK 1, 2, and 3), and p38 kinases (Roux and Blenis, 2004). Growth factors are the primary initiators of ERK1/2 activation, while JNK and p38 activities are stimulated by a number of physical and chemical stressors such as oxidative stress, UV irradiation, hypoxia, and various inflammatory cytokines including TNFα (Chen et al., 2001; Kyriakis and Avruch, 2001). There are three tiers of protein kinases that comprise each family of MAPKs: (1) MAPK; (2) MAPK kinase (MAPKK); and (3) MAPKK kinase (MAPKKK; Figure 3). MAPKKK phosphorylates and activates MAPKK, which then phosphorylates and activates MAPK to then phosphorylate target substrates (Keshet and Seger, 2010).

While ERK1/2 signaling has been implicated in cell proliferation and survival, TNFα activation of JNK and p38 signaling cascades promotes downstream events associated with cell death (Wajant et al., 2003). P38 is a mediator of apoptosis and it does so through several transcriptional and posttranscriptional molecular mechanisms. P38 activation can promote Bax mRNA and protein expression while phosphorylating and deactivating pro-survival Bcl-2 protein, thereby stimulating Bax-mediated mitochondrial apoptosis and cyt *c* release (Porras et al., 2004; Capano and Crompton, 2006; Markou et al., 2009; Owens et al., 2009). Pro-death factors such as Bcl-2-like protein 11 (Bim, a pro-apoptotic regulator), FasL, and FasR have also been positively regulated following p38 MAPK activation to stress stimuli (Hsu et al., 1999; Stephanou et al., 2001; Porras et al., 2004; Cai et al., 2006). Through inhibition of cellular FLIP (c-FLIP) in the DISC of the extrinsic apoptotic pathway, p38 may also trigger Fas-mediated caspase-8 dependent programmed cell death (Tourian et al., 2004). There is some evidence that p38 may also promote apoptosis by blocking ERK1/2 signaling cascades associated with cell survival (Porras et al., 2004). Blocking p38 MAPK activity has been associated with protection against noise trauma, aminoglycoside, cisplatin, radiation and TNFα-induced ototoxicity (Wei et al., 2005; Tabuchi et al., 2010; Bas et al., 2012a,b; Park et al., 2012; Wang et al., 2012a,b; Maeda et al., 2013; Kim et al., 2014; Kurioka et al., 2014; Shin et al., 2014).

JNK has three isoforms: JNK1, JNK2, and JNK3. JNK1 and JNK2 are expressed ubiquitously while JNK3 is primarily localized to cardiac and neuronal tissues. In response to an extracellular stimulus, JNK signaling can promote apoptosis through two distinct mechanisms: one targeting the nucleus and the other targeting the mitochondria. JNK can translocate to the nucleus and phosphorylate c-Jun and other transcription factors such as p53, subsequently promoting expression of pro-apoptotic genes (such as TNFα, FasL, Bak, Bim, and Bax) and blocking transcription of anti-apoptotic genes (such as Bcl-2) (Faris et al., 1998; Budhram-Mahadeo et al., 1999; Eichhorst et al., 2000; Fan et al., 2001; Whitfield et al., 2001; Liu and Lin, 2005; Oleinik et al., 2007; Dhanasekaran and Reddy, 2008; Amaral et al., 2010). The JNK-mediated upregulation of pro-inflammatory and pro-apoptotic proteins and downregulation of pro-survival proteins can provide the necessary substrates for fueling pro-death cellular events.

In addition, activated JNK can phosphorylate a number of proteins that are intimately involved in mitochondrial cell death. By phosphorylating the pro-death proteins Bim and Bad, activated JNK can neutralize Bcl-2 and Bcl-xL protein inhibition while promoting Bax activation of intrinsic apoptotic cell death (Ottilie et al., 1997; Puthalakath et al., 1999; Donovan et al., 2002; Lei et al., 2002; Lei and Davis, 2003; Wang et al., 2007a,b). Furthermore, phosphorylated JNK can indirectly trigger pro-apoptotic Bax-mediated mitochondrial death signaling through truncation of Bid. The constellation of these events results in release of cyt *c*, Smac, and other mitochondrial pro-death proteins into the cytoplasm that promote caspase-dependent and -independent mechanisms of programmed cell death (Tournier et al., 2000; Madesh et al., 2002; Deng et al., 2003; Dhanasekaran and Reddy, 2008).

Although JNK activity is primarily linked to apoptotic cell death, prolonged JNK activation is thought to divert TNFα-induced cell death from apoptosis to necrosis. Elevated levels of ROS can promote prolonged JNK activation, while prolonged JNK signaling can lead to increased mitochondrial production of ROS, creating a positive feedback loop that favors TNFα-induced necrotic cell death (Sakon et al., 2003; Wajant et al., 2003; Ventura et al., 2004).

Similar to p38, JNK signaling is implicated in noise, aminoglycoside, cisplatin, radiation and TNFα-initiated loss of auditory HCs and with a resultant hearing loss. The insertion of an electrode array of a cochlea implant into the cochlea also exhibits effects of JNK and downstream molecular events. Direct inhibition of JNK by either CEP-1347 or D-JNKI-1 (aka AM-111) have demonstrated protection against HC and hearing losses following aminoglycoside, acoustic, and electrode insertion injury to the cochlea (Ylikoski et al., 2002; Wang et al., 2003, 2007a,b; Eshraghi et al., 2006, 2010; Jiang et al., 2006; Suckfuell et al., 2007). Indirect modulation of JNK activity by various drug therapies have been associated with HC and hearing protection (Nakamagoe et al., 2010; Pyun et al., 2011; Shin et al., 2014).

### Oxidative Stress

ROS are free radicals containing oxygen. They are very reactive and in high amounts can damage cells. Extracellular ROS can be generated by activated phagocytes such as neutrophils, monocytes, and macrophages, and play a vital role in host defense against pathogens (Evans and Halliwell, 1999). Intracellular ROS are primarily produced by the cell’s mitochondria (Turrens, 2003). Oxygen-derived free radicals can induce apoptosis and necrosis by peroxidation of a cell’s phospholipid membranes, proteolytic degradation, and DNA damage in mitochondria and in the nucleus (Beckman and Ames, 1997; Kohen and Nyska, 2002).

Oxygen (O_2_) contains two unpaired electrons. Superoxide anion (${\text{O}}_{\text{2}}^{\text{−.}}$) is the product of one electron reduction of O_2_ and is the precursor for several ROS. ${\text{O}}_{\text{2}}^{\text{−.}}$ can react with nitric oxide and produce RNS, such as peroxynitrite, that are also detrimental to cell viability (Beckman and Koppenol, 1996).

The main enzymatic source of ${\text{O}}_{\text{2}}^{\text{−.}}$ is NAD phosphate (NADPH) oxidases, which are located on the cellular membrane of phagocytes. NADPH oxidases can transfer electrons from NADPH to O_2_ to produce ${\text{O}}_{\text{2}}^{\text{−.}}$ (Dworakowski et al., 2006). The mitochondrial electron transport chain (ETC) is the predominant non-enzymatic supplier of ${\text{O}}_{\text{2}}^{\text{−.}}$. The ETC involves several redox enzymes that act in sequence to couple electron transfer to proton translocation across the mitochondrial membrane, creating a transmembrane electrochemical proton gradient. This gradient is crucial for driving ATP synthesis and cellular events that depend on ATP as an energy source. The ETC can leak electrons to O_2_, partially reducing this molecule to ${\text{O}}_{\text{2}}^{\text{−.}}$ (Turrens, 2003). Overproduction of ROS can promote cell death following various stress signals.

Classically, ROS is not associated with the DR (death receptor)-mediated apoptosis; however there is some recent evidence that oxidative stress can turn on extrinsic cell death signaling. This again demonstrates the many levels of communication that occur both upstream and downstream in events of programmed cell death. ROS can activate apoptosis signal-regulating kinase-1 (ASK-1), which is a MAPKKK that can phosphorylate and activate mediators of the JNK and p38 pathways of extrinsic programmed cell death (Nagai et al., 2007). ROS may also promote JNK-mediated extrinsic apoptosis through oxidation and inhibition of MAPK phosphatases that normally suppress JNK activity (Kamata et al., 2005).

Intrinsic cell death can also be affected by oxidative stress. High levels of ROS can initiate nuclear accumulation of FOXO3 (O subclass of the forkhead family of transcription factors) that can trigger up regulation of genes important for cell death such as Bim and Bcl-6 (Hagenbuchner et al., 2012; Sinha et al., 2013). FOXO3 can promote Bim translocation to the mitochondria and knockdown of anti-apoptotic protein Bcl-xL, which can modulate Bax-mediated mitochondrial cell death (Sedlak et al., 1995; Khawaja et al., 2008). It is hypothesized that FOXO3 can regulate the expression and activation of various pro-apoptotic and pro-survival proteins of the Bcl-2 family that can lead to mitochondrial stress, transient opening of the MPT pore, collapse of the mitochondrial membrane potential, release of mitochondrial pro-death proteins into the cytoplasm, and transient increases in ROS from the ETC. This downstream mitochondrial production of ROS can promote a positive feedback loop that converges into apoptosis (Sinha et al., 2013). In this positive response, overproduction of ROS can then promote more oxidation of lipids, proteins, and nucleic acids, disruption of mitochondrial integrity and induction of apoptosis and possibly necrosis. In addition, these free radicals can also oxidize cardiolipin, which results in tBID binding of the voltage-dependent anion channel (VDAC) of the MPT pore and downstream events associated with intrinsic apoptosis (Kagan et al., 2005).

Cochlear tissues produce high levels of ROS in response to a variety of challenges, such as gentamicin, radiation, cisplatin, electrode insertion trauma, and noise exposure (Henderson et al., 2006; Rybak et al., 2007; Choung et al., 2009; Low et al., 2009; Bas et al., 2012a,b). Cells can counteract the effects of oxidative stress through an antioxidant defense system that comprises free radical scavengers and antioxidant enzymes such as reduced glutathione (GSH), superoxide dismutases, and catalase. Insufficient levels of antioxidants such as GSH and vitamins A, C, and E are also associated with oxidative stress. It is the imbalance produced by ROS and antioxidant activity that can help determine the fate of an affected cell. Antioxidants and free-radical scavengers that have demonstrated protection against cochlear injury include iron chelators, manipulation of dietary glutathione, D-Methionine, lipoic acid, N-acetylcysteine, alpha-tocopherol, and m40403 (a superoxide dismutase mimetic), resveratrol, nitro-L-arginine methyl ester (L-NAME) and co-enzyme (Evans and Halliwell, 1999; Ohlemiller et al., 1999, 2000; Huang et al., 2000; Teranishi et al., 2001; Seidman et al., 2003; Nicotera et al., 2004; Samson et al., 2008; Fetoni et al., 2009; Rewerska et al., 2013). Dexamethasone is a synthetic steroid that has demonstrated beneficial effects against several inner ear disorders; dexamethasone can block the expression of ROS as well as reduce the extracellular inflammatory response and block extrinsic cell death signaling through activation of NFkB (Bas et al., 2012a).

## Acoustic Trauma and Auditory HC Death

**Figure 4:**
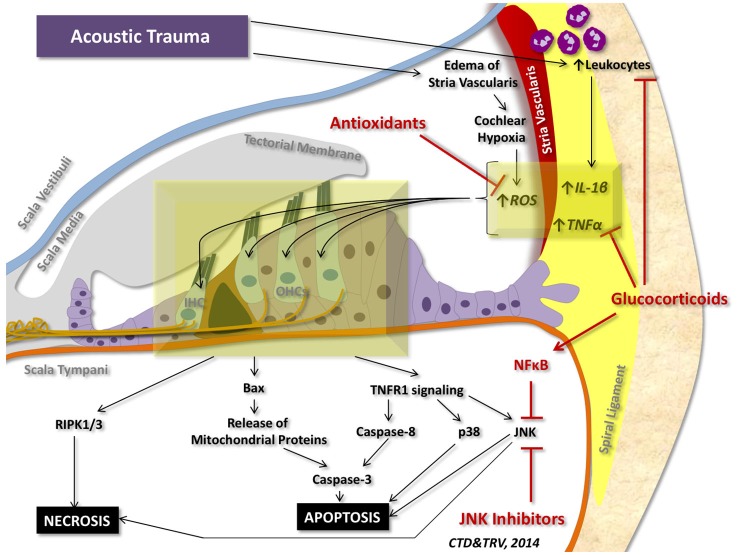
**Cell death signaling following acoustic trauma**. Acoustic trauma can promote edema of the stria vascularis, resulting in cochlear hypoxia, formation of ROS, and oxidative stress. Noise trauma can also stimulate the spiral ligament to express cytokines (such as TNFα and IL-1β) and chemokines that initiate migration of leukocytes to the cochlea; leukocytes will then release a number of other pro-inflammatory factors and free radicals that propagate the inflammatory process. Oxidative stress and pro-inflammatory cytokines can travel to the inner hair cells (IHC) and outer hair cells (OHCs) of the organ of Corti and induce intrinsic and extrinsic apoptotic signaling cascades. Prolonged activation of JNK and induction of RIPK1/3 activity can promote necrotic cell death. Glucocorticoids have demonstrated protection against acoustic trauma, in part by reducing leukocyte migration, decreasing expression of pro-inflammatory factors, and activating NFκB inhibition of JNK signaling. Antioxidants and free radical scavengers can neutralize ROS and downstream effects of oxidative stress. Inhibitors of JNK can also mitigate noise-induced loss of auditory HCs by preventing downstream signaling cascades that lead to apoptosis and necrosis.

Although a number of stressors can initiate pro-inflammatory and pro-cell death signaling within the cochlea, the most studied insult to the inner ear is acoustic trauma. Acoustic trauma initiates a sequence of events within the cochlea that can culminate in apoptosis and regulated necrosis of auditory HCs (Figure 4).

Loud noise exposures produce large displacements of the tympanic membrane and propagate waves of mechanical energy into the inner ear. These intense pulses can produce rapid displacement of cochlear fluid within the inner ear, causing shearing force damage to the OC and basilar membrane, injuring auditory HCs, and altering the endocochlear potential (EP). Acoustic trauma can also initiate inflammation and edema of the stria vascularis and compromise the blood supply to the cochlea (Smith et al., 1985). Since there is minimal redundancy in cochlear blood flow, even transient reductions in cochlear blood flow can result in hypoxia and injury to auditory HCs. Injuries to the stria vascularis and spiral ligament occur following noise trauma, damaging type II and type IV fibrocytes that are important for maintenance of theEP) and lead to permanent hearing loss (Hirose and Liberman, 2003).

Acoustic trauma can lead to ROS release by the marginal cells of the stria vascularis as a result of reductions in cochlear blood flow and cochlear hypoxia (Yamane et al., 1995; Ohlemiller et al., 1999). It is controversial how hypoxia results in oxidative stress; however, it may be related to an increase in oxygen demand by auditory HCs followed by increased aerobic respiration by mitochondria to produce ATP and production of free radical byproducts via the ETC (Yamane et al., 1995; Taylor, 2008). The lateral wall structures of the cochlea also express cytokines (such as TNFα and IL-1β) and chemokines following acoustic trauma, which stimulate migration of leukocytes into the cochlea via the spiral modiolar vein (Keithley et al., 2008). These inflammatory cells will in turn release more cytokines and chemokines as well as produce ROS and RNS that will propagate the inflammatory process in the inner ear.

When TNFα binds to its complementary receptor TNFR1, TRADD and FADD are recruited along with caspase-8. Caspase-8 can activate executioner caspase-3 to promote extrinsic cell death through DNA fragmentation and chromatin condensation via CAD, ACINUS, and HELI-CARD or it can cleave BID into tBID, which can activate Bax-mediated intrinsic, mitochondrial cell death (Liu et al., 1997; Enari et al., 1998; Li et al., 1998; Kovacsovics et al., 2002; Nicotera et al., 2003; Wang et al., 2003; Murai et al., 2008; Jamesdaniel et al., 2011; Infante et al., 2012). Furthermore, loud noise exposure can upregulate TNFα-mediated p38 and JNK signaling in the sensory epithelium of the inner ear that promotes transcription of pro-death genes and Bax-mediated mitochondrial release of apoptotic proteins such as cyt *c* (Wang et al., 2007a,b; Dinh et al., 2008a,b; Infante et al., 2012; Jamesdaniel et al., 2011). Fas-mediated apoptosis also occurs following acoustic trauma (Jamesdaniel et al., 2011).

Oxidative stress from noise exposure can also initiate intrinsic apoptotic cell death in auditory HCs, resulting in mitochondrial release of cyt *c* into the cytoplasm, generation of apoptosomes, and activation of caspase-3 dependent cell death. AIF and EndoG are also released into the cytosol, translocate into the nucleus, and can initiate chromatin condensation and DNA fragmentation of auditory HCs (Nicotera et al., 2003; Yamashita et al., 2004a,b; Han et al., 2006; Henderson et al., 2006).

In addition to apoptosis, acoustic trauma can result in regulated necrosis through RIPK3/RIPK1 activation in rats, that was reversed with necrosis inhibitor necrostatin-1 (Zheng et al., 2014).

Acoustic trauma can also promote swelling and rupture of dendritic terminals of cochlear nerve afferent fibers (Spoendlin, 1971). Intense noise exposure can trigger inner HCs to release significant amounts of glutamate into the synapses with type I fibers of the cochlear nerve. The glutamate overwhelms the postsynaptic glutamate receptors and results in ion influx into dendritic terminals of the cochlear nerve that leads to loss of auditory nerve fibers (Pujol et al., 1990; Kujawa and Liberman, 2009).

Furthermore, intense noise exposure can increase intracellular calcium in auditory HCs and activate calcium-dependent calpains—cysteine proteases that promote proteolysis and breakdown of cytoskeletal and membrane proteins, kinases, phosphatases, and transcription factors (Wang et al., 1999). Subsequently, calpain cleaves and activates calcineurin that promotes Bad-mediated mitochondrial release of apoptotic factors that lead to intrinsic cell death (Le Prell et al., 2007).

Otoprotective drugs can target different levels in apoptosis and necrosis signaling pathways following acoustic trauma (Figure 4). Glucocorticoids can reduce extracellular inflammatory cell trafficking, reduce the level of TNFα expression in spiral ligament fibrocytes, and bind to its glucocorticoid receptors in auditory HCs to activate pro-survival NFkB signaling and inhibit JNK signaling. Additionally, they can reduce oxidative stress, and regulate pro-death and pro-survival gene transcription (Maeda et al., 2005; Tahera et al., 2006; Dinh et al., 2008a,b, 2011; Bas et al., 2012b). Regulation of MAPK signaling can also protect against noise-induced hearing loss by reducing p38 and JNK MAPKs (Tabuchi et al., 2010). In particular, direct and indirect inhibitors of JNK can block JNK phosphorylation of c-Jun and p53 and suppress downstream activation of intrinsic and extrinsic forms of apoptosis and potentially necrosis following acoustic trauma (Sakon et al., 2003; Wang et al., 2003, 2007a,b; Ventura et al., 2004; Coleman et al., 2007; Suckfuell et al., 2007). Lastly, antioxidants and free-radical scavengers can neutralize ROS that is responsible for lipid peroxidation of cell membranes, protein degradation, DNA injury and other mechanisms related to oxidative stress induced apoptosis and necrosis of noise-injured auditory HCs (Seidman et al., 2003; Nicotera et al., 2004; Nordang and Anniko, 2005; Samson et al., 2008; Fetoni et al., 2009; Rewerska et al., 2013).

## Conclusion

Numerous diverse insults to the inner ear can cause auditory HC damage and hearing loss. The evolutionarily conserved apoptotic and necrotic cell death signaling that occurs in auditory HCs is shared among many ototoxic and traumatic stressor events. The most well studied molecular mechanisms behind cell death in auditory HCs involve TNFα signaling, JNK and p38 activation and the effect of high levels of oxidative stress. Although their effects on intrinsic and extrinsic pathways of apoptosis have been studied extensively, there are likely many levels of cross communication between signaling cascades that are still undiscovered. Research in this area is becoming more prevalent, as well as research into mechanisms of regulated necrosis in auditory HCs. A number of otoprotective drug therapies target different levels along this pro-inflammatory pro-death signaling cascade to block downstream events that lead to cell death and promote auditory HC viability and hearing protection.

## Conflict of Interest Statement

The authors declare that the research was conducted in the absence of any commercial or financial relationships that could be construed as a potential conflict of interest.
